# Investigation of the Influence of Moisture Content on Fatigue Behaviour of HPC by Using DMA and XRCT

**DOI:** 10.3390/ma15010091

**Published:** 2021-12-23

**Authors:** Martin Markert, Josef Katzmann, Veit Birtel, Harald Garrecht, Holger Steeb

**Affiliations:** 1 Materials Testing Institute, University of Stuttgart, Pfaffenwaldring 4d, 70569 Stuttgart, Germany; veit.birtel@mpa.uni-stuttgart.de (V.B.); harald.garrecht@mpa.uni-stuttgart.de (H.G.); 2 Institute of Applied Mechanics (CE), University of Stuttgart, Pfaffenwaldring 7, 70569 Stuttgart, Germany; josef.katzmann@mechbau.uni-stuttgart.de (J.K.); holger.steeb@mechbau.uni-stuttgart.de (H.S.); 3 Stuttgart Center for Simulation Science (SC Sim Tech), University of Stuttgart, Pfaffenwaldring 5a, 70569 Stuttgart, Germany

**Keywords:** fatigue behaviour, high-performance concrete, temperature development, moisture content, strain development, dynamic mechanical analysis, X-ray

## Abstract

High-performance concrete (HPC) is a topic of current research and construction projects, due to its outstanding compressive strength and durability. In particular, its behaviour under high-cycle fatigue loading is the focus of current investigations, to further pave the way to highly challenging long-lasting constructions; e.g., bridges or offshore buildings. In order to investigate the behaviour of HPC with different moisture contents in more detail, a mixture of silica sand and basalt aggregate with a maximum grain size of 8 mm was investigated with three different moisture contents. For this purpose, cyclic compressive fatigue tests at a loading frequency of 10 Hz and different maximum stress levels were performed. The main focus was the moisture influence on the number of cycles to failure and the development of concrete temperature and strain. In a further step, only the mortar matrix was investigated. For this purpose, the mixture was produced without basalt, and the moisture influence was investigated on smaller-sized test specimens using dynamic mechanical analysis (DMA) and X-ray computed tomography (XRCT). It was shown that the moisture content of HPC had a significant influence on the fatigue damage behaviour due to the number of cycles to failure decreasing significantly with increased moisture. In addition, there was also an influence on the temperature development, as well as on the strain development. It was shown that increasing moisture content was associated with an increase in strain development. XRCT scans, in the course of the damage phases, showed an increase in internal cracks, and made their size visible. With the help of DMA as a new research method in the field of concrete research, we were also able to measure damage development related to a decrease in sample stiffness. Both methods, XRCT and DMA, can be listed as nondestructive methods, and thus can complement the known destructive test methods, such as light microscopy.

## 1. Introduction

The days when concrete was a simple three-substance mixture of cement, water, and aggregates are over. Today, a variety of concrete additives and admixtures are available for the development of ultra-high-performance concrete (UHPC) and high-performance concrete (HPC). The fatigue behaviour of these concretes is very complex and not fully understood, even after many years of research. Due to missing information on the fatigue damage processes, the related safety design factors are set to be on the safe side. Because the fatigue behaviour of HPC and UHPC is particularly important with regard to service life design equations, constructions using said concretes must be designed very conservatively [1,2]. So far, mainly the influence of external fatigue loads has been studied, especially that of the frequency and amplitude. The latest research has shown that moisture content of the concrete itself; i.e., viscous fluid inside the pore space of HPC and UHPC, has a significant influence on the effective fatigue behaviour [3,4,5,6]. In this context, the term moisture is used in the following to mean the volume of water absorbed by the concrete through different storage conditions. The term moisture thus stands for free water content in the pores, as well as physically bound water. The influence on the temperature development on the fatigue behaviour was discussed in [7,8], as well as in [9]. In addition to the influence of moisture and temperature, the maximum grain size or the concrete composition process also seems to have an influence on the fatigue behaviour. In [10], for example, it was shown that the type of aggregate had an influence on the fatigue strength. An influence of the maximum grain size was found in [8] as well as in [11]. Therefore, there is an effort to increase the fatigue resistance by improving or optimising the concrete composition. For this purpose, the fatigue behaviour of concrete and the associated matrix without coarse aggregate was investigated. In addition to the development of the compressive strength, established damage indicators such as the strain development of concrete under fatigue loading were investigated. In order to observe the damage processes in the interfacial transition zone (ITZ), high-resolution examination methods are required. Nondestructive testing methods such as XRCT cannot characterise inherent phenomena in the ITZ between the mixture aggregates of the different concretes with regard to limitations in (µ)XRCT resolution. In order to be able to characterise these effects on an effective scale, a concrete with a maximum grain size of 2 mm was investigated using dynamic mechanical analysis (DMA). DMA is sensitive to inherent stiffness evolution of the (microfractured) HPC, and probes the sample with small amplitudes. The HPC mixture was investigated and compared both in the undamaged and in the specifically damaged states. This showed that DMA and XRCT could be used to detect and describe the damage evolution. Changes in the E-module and the effects of cracks in the ITZ can be made visible. The investigations of concrete by varying the moisture proved the effect on the number of cycles to failure, strain, and temperature development.

## 2. Materials and Methods

### 2.1. Material and Geometry

The HPC used in this study was the reference mixture developed for the Priority Programme SPP2020 [3,4]. It was designed with the aim to obtain a concrete that simultaneously had as few pores as possible and a high compressive strength. Table 1 gives an overview of the chosen composition for the HPC. The water/cement ratio (w/c) was calculated from the amount of cement (CEM I 52.5 R—SR3 (na) = 500 kg/m³) and water (176 kg/m³) to w/c = 0.35. Two different variants of HPC were investigated. The first variant was HPC8, as described in Table 1, with basalt with up to an 8 mm grain size. The characteristics of the used basalt were determined on 3 cylinders of d = 150 mm and h = 300 mm [12]. A compressive strength $f_{cm}$ of 326 MPa and a Young’s modulus $E_{m}$ of 99.8 GPa were determined. The Poisson’s ratio and the shear modulus were not determined. In the literature, [13] the compressive strengths and elastic moduli of basalt have been compared for many different geographical sources. The values for compressive strength $f_{cm}$ differed between 81 MPa and 487 MPa, while the values for Young’s modulus were between 69 GPa and 127.9 GPa. For the 2 mm variant (HPC2), no basalt was added. For the fatigue tests of HPC8, cylindrical specimens with a diameter of 60 mm and a height of 180 mm were prepared. To investigate the influence of moisture, three different storage conditions were used for the HPC8. The storage conditions of test series C (climate chamber storage) were defined as a temperature of 20 °C and 65% relative humidity after one week of underwater storage.

For the storage condition D (dry specimen), the specimens were stored as series C and dried at 105 °C after 56 days. The test specimens of series UW (underwater) remained underwater until experimental investigation. The exact storage conditions and the resulting concrete moisture can be found in Table 2. To determine the concrete moisture, the cylinders were dried in an oven at 105 °C for at least 14 days until no mass loss was measurable. The amount of water was then calculated by weighing the samples before and after oven drying. A residual water quantity of 0.1% was assumed for dried specimens.

**Figure 1:**
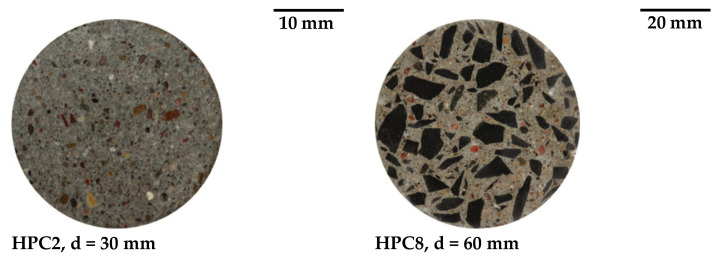
Concrete structure (polished surfaces) depending on the maximum grain size.

For the experiments with HPC2, cubes with an edge length of 100 mm were prepared, from which cylinders with a diameter of d = 30 mm and a height of 75 mm were drilled out. For more details on sample preparation, see Section 2.2. The difference in concrete structure between the both mixtures (HPC2 and HPC8) can be seen in Figure 1.

### 2.2. Sample Preparation and Application

**Figure 2:**
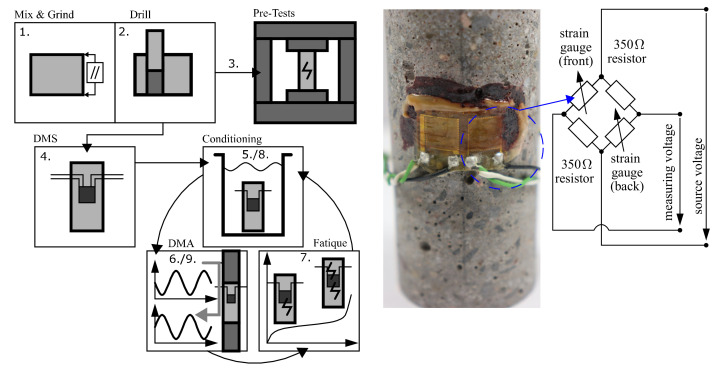
Schematic diagram of the experimental workflow (**left**). Example of measuring bridge wiring (**right**).

A schematic diagram of the major workflow is depicted in Figure 2 (left). First, the HPC was mixed and cast into cuboid blocks (1). Afterwards, these blocks were ground plane-parallel, and then cylinders were drilled out of them (2). Some of these cylinders were used for the preparational static and dynamic failure tests (3). The other cylinders were equipped with strain gauges (Typ K-CXY3 0°/90°-T-Rosette 6 mm from HBM), with wiring as shown Figure 2 (right) (4). The strain gauges were glued on with the 2-component adhesive X60 from HBM. Afterwards, they were conditioned for testing (5). In this case, they were left in water for about 36 h, and then subjected to DMA (6) in their intact state (‘0′). In the next step, fatigue damage also was induced inside these samples using dynamic failure tests (7), leaving them in the microcracked state (‘C’). In this state, they were again conditioned and tested by DMA (8 and 9). For the specimens with a diameter of 60 mm from HPC8, the cylindrical specimens were cut to length, and the top surfaces were ground plane-parallel, polished, and then stored at 20 °C and 65% relative humidity (r.h.). Immediately before testing, the curved surface areas of the cylinders were sealed with a water vapor diffusion-tight aluminium composite foil (profiTHERM) to prevent drying out during the test.

### 2.3. Test Setup—Fatigue Test

**Figure 3:**
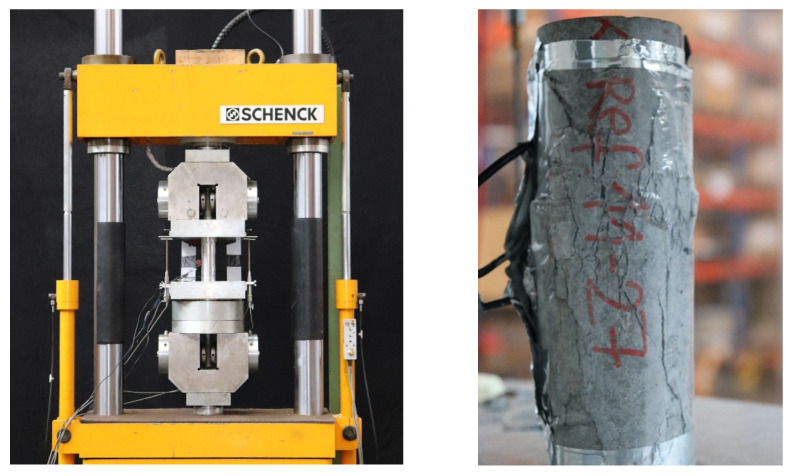
Test setup fatigue test of Schenck PC 400 N (**left**). Specimen of HPC8 with a maximum grain size of 8 mm after fatigue test (**right**).

The fatigue tests were conducted on two different servo-hydraulic Schenck testing machines (Schenck Hydropuls PC 400 N and PC 400 M/So, Germany) with a maximum load capacity of 400 kN (d = 60 mm) or 250 kN (d = 30 mm). Each test was operated load-controlled. The 400 kN testing machine and the test setup are shown with cylinder (d/h = 60/180) in Figure 3 (left). To minimise unintended bending of the test specimen due to imperfection of the plane-parallel top and bottom sides of the cylinders, a load-transfer plate with a cup and ball bearing was used. In order to keep the moisture as unchanged as possible during the tests, the samples were packed vapour-tight in foil. During the tests, the number of load cycles, the applied forces, the deformation of the specimen, and the temperature evolution were measured. The surface temperature in the middle of the samples and the room temperature were measured using temperature sensors. The deformation was measured by four inductive displacement transducers WA 1–4 adapted between the load transfer plates on every side.

The strain was calculated from the measured deformations under the assumption of constant strain over the height of the specimen. The temperature was measured, in addition to the ambient temperature, at mid-height on the surface. During the tests with the HPC8, the temperature was measured at 3 points on the surface, as well as the room temperature and the temperature of the steel plate. The exact configuration was taken from [3]. A fractured test specimen with an 8 mm maximum grain size (HPC8-N) after the fatigue test is shown in Figure 3 (right).

### 2.4. Experimental Programme

All tests were carried out with a sinusoidal loading at a constant frequency of 10 Hz, to be able to compare them with other projects involved in the Priority Programme SPP2020 [3,4]. The selected frequency was therefore sufficiently fast to achieve a sensible number of load cycles within a reasonable time period. This can usually be achieved well with servo-hydraulic testing machines. The normalised minimum stress was S_u_ = σ_u_/f_c,cyl_ = 0.05, and the normalised maximum stress S_o_ = σ_o_/f_c,cyl_ was varied according to Table 3. Regarding the variation of the upper stress level, it was to be verified whether the influence applied to all stress levels. In this case, f_c,cyl_ was the static compressive strength (Table 4), and σ_o_/σ_u_ was the upper/lower stress, respectively. Tests with >2.75 million load cycles were treated as runouts. The specimens of HPC8 with a diameter of 60 mm were examined for their fatigue resistance via the number of cycles to failure, the strain, and the temperature development. The HPC2 specimens were stopped at the different damage phases (Section 3.3) in order to be able to examine them by using DMA and XRCT. This allowed the undamaged status to be compared with the damaged status. The test programme for all series is shown in Table 3.

### 2.5. Dynamic Mechanical Analysis Setup

**Figure 4:**
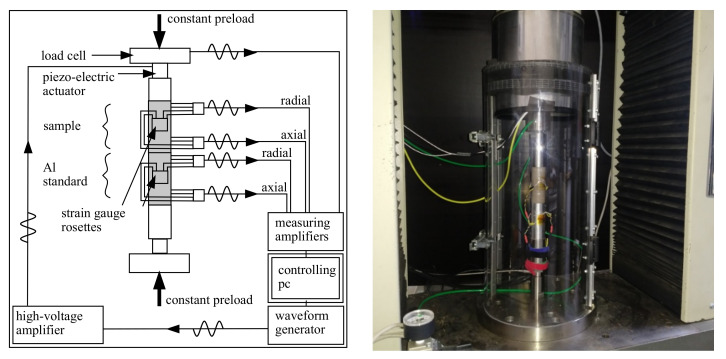
Schematic diagram of the DMA setup (**left**). Picture of DMA setup (**right**).

A schematic diagram of the DMA setup is shown in Figure 4. Similar setups for DMA with axially applied force have been used in rock core studies to characterise sedimentary rocks. A detailed overview of these and other DMA setups used in geophysics can be found in [14,15]. For the DMA setup (Figure 4), a precalibrated aluminium (Al) cylinder was used for the force measurement. For harmonic excitations, a high-voltage piezo-electric actuator (Physik Instrumente P-235.0, Karlsruhe, Germany) was used; see also [15,16,17]. The setup (Figure 4) was mounted in a static universal testing machine (Schenck RSA 100, Germany, refurbished with a digital DOLI EDC controlling unit). For the static testing machine, a static area preload was applied onto the sample using a metallic stamp. The piezo-electric actuator was placed at the top of the sample. The actuator transformed electrical voltages between 10 and 1000 V into displacements with amplitudes up to 35 μm. The amplitude of the displacements depended on the static preload, the excitation frequency, and the overall stiffness of the setup. In general, the relation between input voltage and actuator displacements was highly nonlinear. The actuator input was initially offset by 500 V and then excited sinusoidally. For this purpose, a sinusoidal voltage signal was transmitted by an arbitrary waveform generator (AWG, Agilent 33500B, Santa Clara, CA, USA) and amplified by a high-voltage power amplifier (Physik Instrumente E-482, Karlsruhe, Germany). The maximal peak-to-peak amplitude for the harmonic signal amounted to 10 Vpp, which corresponded to a 1000 V amplitude at the piezo-electric actuator (40 dB amplification). The samples and Al standard were equipped with two strain gauge biaxial (tee) rosettes. From these, each two longitudinal and transversal gauges, respectively, were wired to a Wheatstone diagonal half-bridge, as shown in Figure 2. Consequently, the longitudinal and transversal strains were averaged to enhance the signal-to-noise-ratio (SNR). Apart from the strain measurements, the temperature near the sample was measured with a PT1000 (Resistance Temperature Detectors, RTD) temperature sensor. The Al standard that was used for high-frequency force measurements was calibrated at low frequencies with the machine internal load cell (Schenk 100 kN, Germany).

Strain, force, and temperature signals were amplified and digitised by three universal measuring amplifiers (HBM QuantumX, MX410/B, and MX440, Germany, respectively). The AWG and the measuring amplifiers were computer-controlled by MATLAB scripts. In the postprocessing step, the complex coefficients of the strain and force signals were determined by averaging the results from a sliding-window fast Fourier transformation (FFT). From the complex coefficients, the frequency-dependent complex Young’s modulus E and Poisson’s ratio ν, generalized here as material parameters H, were calculated as magnitude |H| and intrinsic attenuation 1/Q_H_: = Im(H)/Re(H), according to [18]. Im(H) and Re(H) were the imaginary and the real parts of the complex material parameters H, respectively [19]. Note that 1/Q_H_ was also denoted as an inverse quality factor or loss factor. To measure the frequency-dependent properties via frequency sweeps, an appropriate preload and excitation amplitude had to be chosen first, both from pretests, which are commonly denoted as amplitude sweeps. The preload strain was defined low enough to prevent (further) damage, while simultaneously being high enough to guarantee an appropriate SNR. The appropriate excitation amplitude was chosen from the results of previous amplitude sweeps to achieve a good SNR and, at the same time, to minimise the dependency of amplitude; i.e., staying in the linear regime for the measured viscoelastic properties. For these amplitude sweeps, the AWG signal excitation amplitude was varied in a logarithmic range between 0.553 and 5.411 Vpp (with an additional 40 dB amplification through the power amplifier). The resulting effective material properties were then evaluated over the samples’ axial strain amplitude.

### 2.6. X-ray Computed Tomography (XRCT) Setup

**Figure 5:**
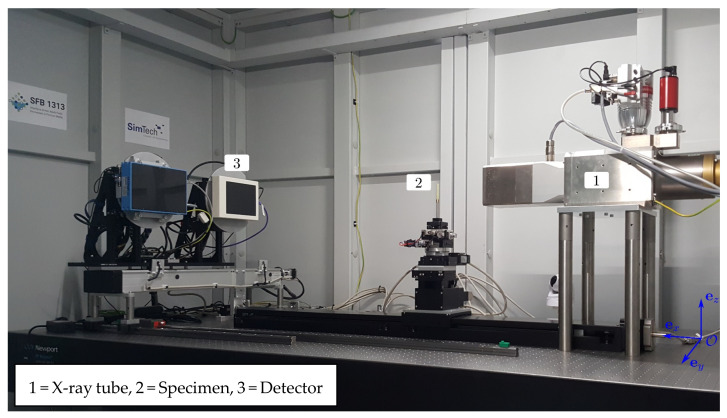
XRCT setup.

For the XRCT scans discussed in the remainder of this paper, a modular laboratory cone-beam XRCT setup was used for 3D microstructural characterisation. Fundamental details of the XRCT method are out of the scope of the present discussion and can be found elsewhere; e.g., [20] or [21]. A recent, detailed review of XRCT and its application to cementitious materials is given in [22]. A detailed description of the in-house developed modular “open” µXRCT system is shown in Figure 5. A technical description, including details of the applied µXRCT workflow from projection acquisition to image reconstruction and postprocessing, can be found in [23,24].

## 3. Results

**Figure 6:**
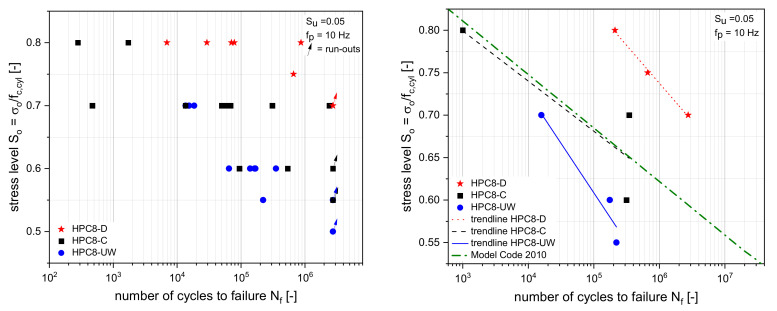
Number of cycles to failure of HPC8 (**left**). Average values of the number of cycles to failure and the corresponding trend lines of HPC8 (**right**).

**Figure 7:**
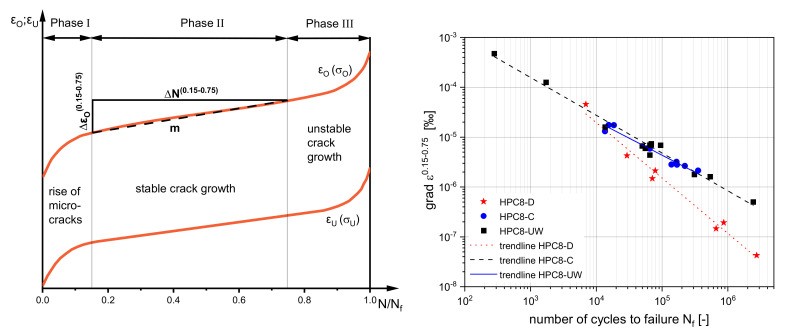
Schematic strain development and division into three phases (**left**). Gradient of strain in damage phase II and the associated trend lines of HPC8 (**right**).

**Figure 8:**
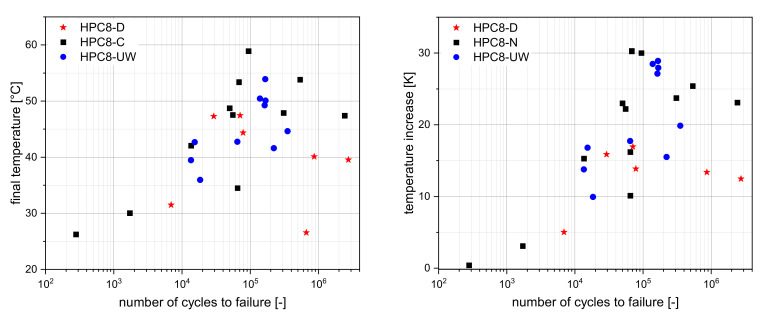
Final temperature (**left**). Temperature increase (**right**).

**Figure 9:**
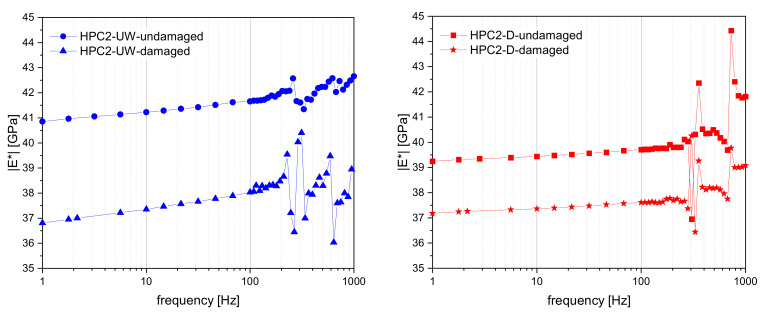
Frequency-dependent Young’s modulus (average of three): HPC2-UW (**left**); HPC2-D (**right**).

**Figure 10:**
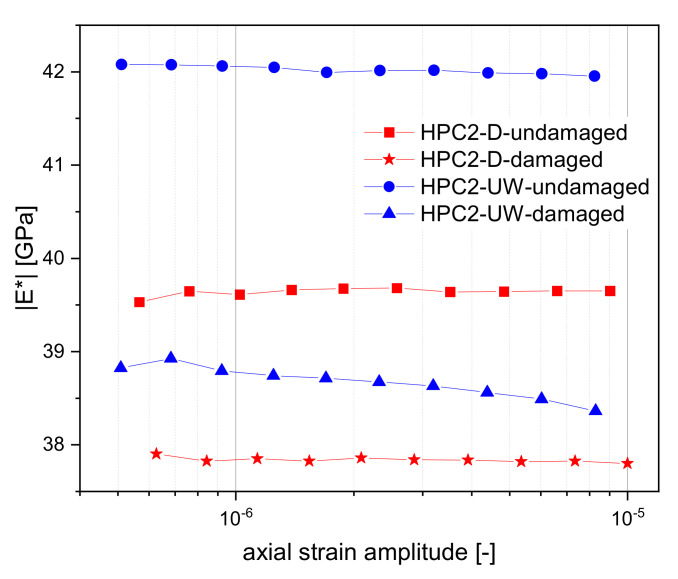
Amplitude-dependent Young’s modulus at 100 Hz (average of three samples).

**Figure 11:**
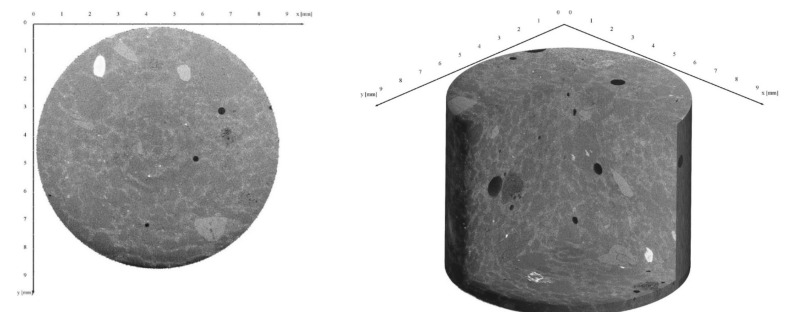
XRCT scan of HPC2-D in phase 0 (undamaged).

**Figure 12:**
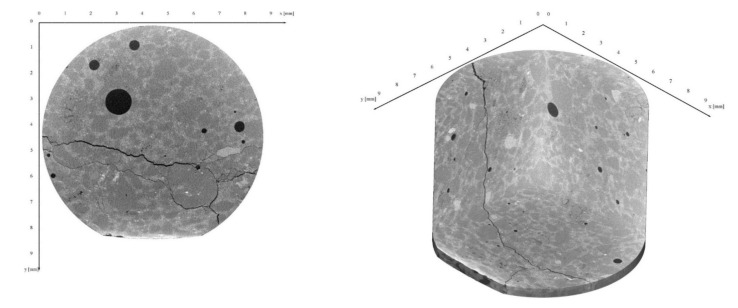
XRCT scan of HPC2-D in phase III.

The results of the investigations are shown in the following subsections. Firstly, the static compressive strengths of HPC8 and HPC2 are given (Table 4), as well as the results of the fatigue tests of HPC8 (Figure 6). Then, the strain and temperature development are analysed in more detail (Figure 7 and Figure 8). Furthermore, the results of the DMA and XRCT investigations of HPC2 are shown in Figure 9, Figure 10, Figure 11 and Figure 12, as well as in Table 5.

### 3.1. Static Compressive Strength

For the calculation of the maximum and minimum normalised stresses S_o_ und S_u_, the static compressive strength f_c,cyl_ was additionally determined for at least three cylindrical specimens before each test series was performed. The average compressive strength values and the standard deviation are shown in Table 4.

For all three HPC8 series, several concrete batches were produced. Therefore, the smallest average value, as well as the largest average value, are given in Table 4. For fatigue testing, it is beneficial if the static compressive strength within a batch does not have large deviations from the average value, because the corresponding stress levels are calculated from the average static compressive strength. The moisture of the concrete was found to have more of an influence on the compressive strength of the 30 mm diameter specimens than on the 60 mm diameter specimens. While the compressive strengths of the 60 mm specimens were similar regardless of the moisture content, there was a significant reduction in the compressive strength of the smaller specimens due to the moisture.

### 3.2. Number of Cycles to Failure

The results of the fatigue tests of HPC8 with different moisture contents according to Table 3 are given in Figure 6. The figures show the results of many different batches. Therefore, the number of cycles to failure was plotted in log scale versus the normalised maximum stress S_o_. All tests were performed with a minimum stress S_u_ = 0.05. Depending on the moisture content, the results for HPC8 are plotted in black, blue, and red colours. The tests that were stopped and removed after 2.75 million load cycles are marked with an arrow. All three moisture contents (HPC8-D ≈ 0.1%; HPC8-C ≈ 4.1% HPC8-UW ≈ 5%) reached the stopping criterion at very different stress levels. The HPC8-UW had already reached the stopping criterion at a stress level of S_o_ = 0.5, while the HPC8-C stopped at S_o_ = 0.55, and the HPC8-D stopped at S_o_ = 0.7. It can thus be stated that the stress level decreased with increasing moisture. Therefore, it was assumed that the moisture led to accelerated fatigue damage. It was noticeable that there was a large scattering of results at the respective upper stress level. This influence could mainly be attributed to the scattering of the static compressive strength in each batch. Due the scattering of results, the average cycles to failure were calculated for each stress level and each test series, and are plotted in Figure 6 (right). Additionally, a linear regression function was determined to describe the fatigue behavior by using the function $f=m\times log\left(N\right)+b$ for each of the three test series. The implementation of a bilinear function, as suggested in Model Code 2010 [25], was not possible due to the small amount of test results with n > $10^{6}$ cycles. For the linear regression, the runouts were not used. The three trend lines in Figure 6 (right) are relatively parallel to each other. All three trendlines supported the assumption that higher concrete moisture values led (blue and black) to early failure compared to dried concrete (red). This influence of moisture was also observed in [3,4,5,6].

In addition to the three trend lines, the line from the Model Code 2010 was included in Figure 6 (right). It can be seen that the results with the climate chamber storage corresponded to the Model Code 2010.

### 3.3. Strain Development as a Damage Indicator

Strain development is known as one of the best indicators of damage [26]. For concrete, the strain development showed a characteristic S-shaped curve in general. This curve can be divided into three phases [26,27,28]; see Figure 7 (left). This damage progression depended on the applied stress levels, as well as the moisture content [3]. In order to be able to better compare the different tests with each other, the gradient m of the strain in phase II was examined and compared. For this purpose, the strain ($\Delta \varepsilon_{O}$) within 15% and 75% of the number of cycles to failure (ΔN) was analysed.

(1) $$m\left(\varepsilon_{O}^{0.15-0.75}\right)=\left(\frac{\Delta \varepsilon_{O}^{0.15-0.75}}{\Delta N^{0.15-0.75}}\right)$$

The runout tests were not evaluated for this purpose. Figure 7 (right) shows the grades of phase II together with the respective ultimate load cycles on a double logarithmic scale. It can be seen that the gradient m was nevertheless on one line, even though the experiments were very scattered. In addition, the moisture content seemed to have an influence to some smaller extent. While the difference between the HPC8-UW and the HPC8-C was hardly noticeable, the difference to the HPC8-D was clearly recognised.

### 3.4. Temperature Development

During the tests, self-induced heating of the specimens was measured in all tests as a result of the applied fatigue loading. This effect is known in the literature [8], and is explained by internal friction. The measured temperature rises mostly in the middle of the test specimen. This effect can be attributed to heat dissipating at the contact surfaces between the steel plate test specimens. Therefore, the temperature effect can play a significant role in the number of cycles to failure [8,29], and it is important to perform a detailed analysis of the temperature evolution. The fact that the moisture has an influence on the heating rate was already investigated in more detail in [3]. There, it was shown that the temperature evolution was similar to the strain evolution, and could therefore be seen as a further damage indicator in addition. For these investigations, the focus was on the maximum temperature and the maximum temperature rise. For this purpose, only those tests were used in which failure occurred.

Figure 8 (left) shows the maximum final temperature (in °C) versus the number of cycles to failure, while Figure 8 (right) shows the temperature increase DT (in K). It can be clearly seen that the maximum heating depended on the moisture and the amount of loading cycles. Since the cycles to failure depended on the upper stress level, the temperature evolution also depended on the applied stress levels. It followed that the temperature tended to increase with an increasing number of cycles to failure, irrespective of the moisture. It also became clear that the temperature of dried concrete (HPC8-D) rose much less than the temperature of concrete with moisture (HPC8-C and HPC8-UW). Due to the strong heating of the samples, the influence on the static compressive strength must be considered in more detail [30,31].

### 3.5. DMA—Dynamic Mechanical Analysis

In the following, the results of the small-strain DMA investigations are shown; these were averaged from three samples each (initial condition as damaged). The HPC2-UW and the HPC2-D were analysed and interpreted. Figure 9 shows the Young’s modulus as a function of the test frequency. Figure 9 (left) shows the averaged results from the wet samples (HPC2-UW), whereas Figure 9 (right) shows the results of the HPC2-D. Both results show that the Young’s modulus was significantly larger in the initial state (undamaged) than in the damaged state. This was expected, as the effective Young’s modulus decreased in the course of fatigue damage. Furthermore, it can be seen that the Youngs’s modulus in the undamaged HPC2-UW samples was larger than in the HPC2-D specimens. This effect was due to the presence of water within the sample. After fatigue damage, the Young’s modulus of both HPC2 specimens was similar. It could be further observed that the effective Young’s modulus depended on the applied frequency. The (positive) dispersion effect was higher for HPC2-UW than for HPC2-D. The increase in dispersion for wet HPC samples could be explained by the role of the viscous fluid within the HPC, which contributed to intrinsic attenuation, and therefore to dispersion. Figure 9 shows a rather large frequency response in the DMA investigations. It could be observed that some system-dependent resonance frequencies above 100 Hz (close to 200–300 Hz and at around 700 Hz). In such DMA tests, resonance frequencies were always expected. For the material properties, mainly the domain lower than 200 Hz, was interesting, in that the fatigue damage behaviour could be well characterised with the help of the DMA and the small load amplitude.

The results for the absolute value of the Young’s modulus in the amplitude domain (“amplitude sweeps”) at a 100 Hz testing frequency in dry (red) and wet (blue) HPC2 are shown in Figure 10. The most important result of this investigation was the rather linear behaviour of properties, especially for the undamaged samples. Especially at applied strains >$10^{-6}$ the results were mainly amplitude-independent with an improved SNR. Damaged samples seemed to be more sensitive to amplitudes compared to undamaged samples. Nevertheless, the amplitude range of $10^{-6}$ to $3\times 10^{-6}$ showed a good compromise between a linear response and a good SNR. It can be seen that in the wet condition, the concrete had a higher Young’s modulus than in the dried condition. This applied to both the damaged and the undamaged state. It can be seen that the Young’s modulus of HPC2-UW decreased from about 42 GPa in the undamaged state to about 38.5 GPa in the damaged state. The Young’s modulus of HPC2-D decreased from 39.5 GPa in the undamaged state to just below 38 GPa in the damaged state. The difference between the undamaged and the damaged state was greater for HPC2-UW than for HPC2-D. This also showed that the Young’s modulus of HPC2-UW and HPC2-D were almost the same in the damaged state.

### 3.6. X-ray Computed Tomography Scans

For the scans using µXRCT, several test specimens were scanned in the undamaged state and in the different damage phases. The scanning area was located in the centre of the sample. Figure 11 shows a scan of HPC2-D in the undamaged state (phase 0). On the left side, a segment can be seen; while on the right side, a tube without a segment is shown. From the two scans, it is easy to differentiate the (cement) matrix and the sand or pores. The higher the density of the material, the lighter it was; therefore, air pores were completely black. There are no obvious cracks in either of the two images shown in Figure 11. Figure 12, on the other hand, shows two scans of a sample of HPC2-D in the damaged state (phase III). This was not the same sample shown in Figure 11. The cracks are clearly visible in both pictures shown in Figure 12. It is visible that the cracks followed the aggregates (here: sand) and passed through the whole scan section in all directions. In phases I and II, cracks could also be seen around aggregate interfaces. The number of cracks, as well as the crack width, increased in the course of the degradation, which is listed in Table 5. It can be seen that the cracks were along the grain boundaries, and consequently in the ITZ. In the case of very large pores, the cracks passed through the pores, while smaller pores remained unnoticed in the crack progression.

## 4. Conclusions

In order to investigate the influence of moisture content on the fatigue behaviour of HPC, two different concrete mixtures were stored in three different environmental conditions and then subjected to cyclic loading. All tests were carried out at a constant frequency of 10 Hz and with a variation of the upper stress level. The strain evolution in the damage phase II of the fatigue experiments correlated with the achieved number of cycles to failure.

An evaluation method was presented that reduced the large scatter range in the plots of failure cycles versus applied load or versus temperature development. A concrete with two different maximum grain sizes was investigated: HPC8, with a maximum grain size of 8 mm; and HPC2, with a maximum grain size of 2 mm. Due to the removal of the basalt aggregate, only the mortar matrix with silica sand was used for HPC2. By stopping the cyclic fatigue tests before failure, it was possible to produce samples of concrete in the respective damage phases. This allowed for a subsequent µXRCT-based characterisation step of predamaged samples. As the method of DMA was nondestructive and was operating in the small strain regime, it could be ideally combined with µXRCT investigations. The collected results are summarised here:

- The different storage conditions caused a different moisture content in the pore space of the concrete. Furthermore, the moisture had a negative effect on the fatigue resistance. At the same applied stress level, the fatigue resistance decreased as the moisture of the concrete increased.
- An influence of the moisture content on the evolution of strains could be observed. For the dry case, the increase in strains per load cycle was smaller than for the water-wet samples.
- The moisture content affected the concrete temperature evolution during the fatigue test. Considering the fact that the compressive strength of concrete is characterised by a temperature dependence, an indirect influence of the compressive strength during the fatigue test could be concluded from the observed moisture–temperature coupling.
- It was possible to detect cracks inside the concrete test specimens with the presented XRCT test setup. The cracks in the different damage phases increased and became larger due to cyclic loading.
- The cracks followed the interface between the cement matrix and the aggregates (sand). It was assumed that the cracks caused by cyclic loading (in the fatigue test) were spread out along the grain boundaries.
- The small-strain DMA method allowed for the (visco)-elastic characterisation of the damage state of the material with high accuracy.
- The damaged state could be characterised by a significant decrease in the value for the effective Young’s modulus compared to the initial state.
- Furthermore, a (dispersive) increase in the Young’s modulus over frequency for each sample was found. This dispersion effect was even more pronounced for wet samples.
- The amplitude sweeps, characterised at a frequency of 100 Hz, showed that the damage could be clearly detected in the measured values of the Young’s modulus.

In summary, it can be stated that moisture had a significant influence on the number of cycles to failure, strain, and temperature evolution. The effect of moisture and temperature in particular should be investigated in further detail in future studies. For this purpose, additional loading frequencies and different concrete mixtures should be considered. The two nondestructive methods (DMA and XRCT) presented in this paper showed that they can be used as characterisation techniques for concrete to attain further information on the damage process.

## Figures and Tables

**Table 1:** Composition of HPC with w/c = 0.35.

Component (–)	Density (kg/dm³)	Amount (kg/m³)
CEM I 52.5 R—SR3 (na)	3.094	500
Quartz Sand H33 (0/0.5 mm)	2.70	75
Sand 0/2	2.64	850
Basalt 2/5	3.06	350
Basalt 5/8	3.06	570
Polycarboxylatether (PCE) Superlasticizer	1.05	4.25
Stabilizer	1.10	2.42
Water	1.00	176

**Table 2:** Storage conditions and concrete moisture of the HPC8.

Series	Day 1	Days 1–7	Days 8–56	Days 56-Test	Before Testing	Moisture Content %
D (Dry Specimen)	Strip the formwork	Underwater	20 °C/65%	105 °C	Wrap	~0.1
C (Climate Chamber Storage)			20 °C/65%	20 °C/65%		4.0
UW (Underwater Storage)			Underwater	Underwater		5.1

**Table 3:** Overview of the test programme.

Series	Storage Condition	S_o_	S_u_	Test Frequency (Hz)
HPC8	D	0.70/0.75/0.80	0.05	10
HPC8	N	0.55/0.60/0.70/0.80	0.05	10
HPC8	UW	0.50/0.55/0.60/0.70	0.05	10
HPC2	D	0.70	0.05	10
HPC2	UW	0.70	0.05	10

**Table 4:** Compressive strengths of the different types of coating.

HPC Variation	HPC8	HPC8	HPC8	HPC2	HPC2
Diameter/height	60/180	60/180	60/180	30/75	30/75
Storage condition	D	C	UW	D	UW
Static compressive strength f_c,cyl_ (MPa)	98.4–104.0	95.4–103.7	106.3–109.9	97.7	88.0
Standard deviation (MPa)	3.63–7.50	3.70–7.36	4.55–7.31	5.29	8.00

**Table 5:** Development of the cracks.

Phase 0 (Undamaged)	Phases I–II	Phase II	Phases II–III	Phase III
No cracks	Cracks w~20–30 µm	Cracks w~30–45 µm	Cracks w~50–80 µm	Cracks w~20–50 µm
	Fine hairline cracks along grain interfaces	Cracks along grain interfaces, through larger pores	Extensive cracks along grain interfaces, through larger pores	Extensive cracks at grain interfaces

## Data Availability

Data available upon request.
